# ADME prediction with KNIME: *In silico* aqueous solubility consensus model based on supervised recursive random forest approaches

**DOI:** 10.5599/admet.852

**Published:** 2020-08-07

**Authors:** Gabriela Falcón-Cano, Christophe Molina, Miguel Ángel Cabrera-Pérez

**Affiliations:** 1 Unit of Modeling and Experimental Biopharmaceutics. Centro de Bioactivos Químicos. Universidad Central “Marta Abreu” de las Villas. Santa Clara 54830, Villa Clara, Cuba; 2 PIKAÏROS S.A., 31650 Saint Orens de Gameville, France; 3 Department of Pharmacy and Pharmaceutical Technology, University of Valencia, Burjassot 46100, Valencia, Spain; 4 Department of Engineering, Area of Pharmacy and Pharmaceutical Technology, Miguel Hernández University, 03550 Sant Joan d'Alacant, Alicante, Spain

**Keywords:** Quantitative Structure-Property Relationship (QSPR), KNIME, aqueous solubility, ADME, machine learning, Random Forest, supervised recursive selection

## Abstract

In-silico prediction of aqueous solubility plays an important role during the drug discovery and development processes. For many years, the limited performance of in-silico solubility models has been attributed to the lack of high-quality solubility data for pharmaceutical molecules. However, some studies suggest that the poor accuracy of solubility prediction is not related to the quality of the experimental data and that more precise methodologies (algorithms and/or set of descriptors) are required for predicting aqueous solubility for pharmaceutical molecules. In this study a large and diverse database was generated with aqueous solubility values collected from two public sources; two new recursive machine-learning approaches were developed for data cleaning and variable selection, and a consensus model based on regression and classification algorithms was created. The modeling protocol, which includes the curation of chemical and experimental data, was implemented in KNIME, with the aim of obtaining an automated workflow for the prediction of new databases. Finally, we compared several methods or models available in the literature with our consensus model, showing results comparable or even outperforming previous published models.

## Introduction

Aqueous solubility is one of the most important physicochemical properties determined during the drug discovery and development processes [1]. It is considered a relevant parameter during ADME (Absorption, Distribution, Metabolism and Excretion) studies [2], and it is a key factor that can affect oral absorption and bioavailability of drugs [3]. Considering that approximately 40 % of drugs on the market and about 75 % of compounds in development have a poor aqueous solubility, early identification of this property should reduce failures in the pharmaceutical development process [4].

Several experimental strategies have been applied to determine the aqueous solubility of compounds, such as variations of the shake-flask method and, more recently, the CheqSol approach [5]. However, the determination of experimental solubility proves to be difficult, time-consuming and too expensive, or unrealistic to test thousands or millions of compounds used in high throughput screening (HTS) [6]. In this respect, the *in-silico* prediction of aqueous solubility by Quantitative Structure-Property Relationship (QSPR) has been widely used in the early stage of the drug discovery and development process [2]. Several QSPR models for predicting aqueous solubility have been developed in recent decades, but the performance of some of these models on various solubility datasets has demonstrated the poor reliability of the prediction methods [2,5,7,8]. One of the main reasons for these results is that published methods are derived from different data sources, for which the root-mean-square errors (RMSE) are around 0.6-0.7 log *S* units [9]. However, some studies suggest that the poor accuracy of solubility prediction is not related to the quality of the experimental data and that more accurate methodologies (algorithms and/or set of descriptors) are required [9,10]. Recently, some machine learning (ML) algorithms such as random forests (RF), support vector machines (SVM), k-nearest neighbors (k-NN), and convolutional and recurrent networks have been applied for aqueous solubility prediction, and their performance matches or outperforms the previous results obtained [11–15].

The Konstanz Information Miner (KNIME) is a free and public software tool that has become one of the main analytical platforms for innovation, discovery of the hidden nature of data and prediction of new features [16]. KNIME, through the implementation of interconnected nodes, integrates several components of machine learning and data mining, which can be easily used in chemistry, biology, drug design and recently in the prediction of ADME properties [17,18]. The flexibility of workflows developed in KNIME to include different tools allows users to read, create, edit, train and test ML models, greatly facilitating the automation of predictions and application by any user.

Taking into consideration the limited predictive performance of many of the published solubility models, the main goal of this study is twofold: the development of an innovative QSPR model using new recursive algorithms for data and variable selection in machine learning approaches, and the implementation of an automatic workflow for a better prediction of aqueous solubility during the early stages of drug discovery and development.

In order to achieve the goal, a large database of aqueous solubility and a new recursive machine learning algorithm for data cleaning and variable selection were used. The application of a consensus regression model and a classification model, using a Random Forest approach, showed good predictive performance in comparison to different models published in the recent literature. Finally, a workflow was designed to automate the prediction of aqueous solubility of new databases, starting from chemical structure.

## Methods

### Computational tool

The open source software KNIME version 4.0.2 [19], its free complementary extensions "*KNIME Base Chemistry Types*" and the "*KNIME Chemistry Add-ons*", were used in this study [20]. Several node extensions such as "*RDKit KNIME Integration*" and "*Indigo KNIME Integration*" were applied for the curation of the databases and the conversion of the chemical structures to different formats, respectively [21,22]. For the generation of molecular descriptors from structures, the “*Descriptor*” node from “alvaDesc” extension was employed [23]. AlvaDesc 1.0.16 is available with academic or commercial licenses, which can be obtained by requesting a quote online (registration required) or by contacting them directly by email (chm@kode-solutions.net).

Other nodes, belonging to KNIME "*Analytics*" and "*Manipulation*" extensions, were used opportunely for the transformation, processing and modeling of data, according to the requirements of the automated workflow.

### Database of aqueous solubility

To develop *in-silico* solubility models, two large publicly accessible databases with experimental values of thermodynamic aqueous solubilities (log *S*, *S* in units of mol/L) were selected. The first database (AqSolDB) was generated by Sorkun *et al.* from nine publicly available datasets and included 9982 liquids and crystalline solid compounds, filtered by temperature for a range of 25 ± 5 °C [24]. The total solubility range of this database is more than eleven orders of magnitude (-9.7 to + 2.1). AqSolDB is openly accessible at the Harvard Dataverse Repository (https://doi.org/10.7910/DVN/OVHAW8). The second database was published by Cui *et al*. and consists of 9943 molecules with experimental solubility values measured at room temperature [12], and its solubility range was more than nineteen orders of magnitude (-18.2 to +1.7, log units).

### Data curation

**Figure 1.**
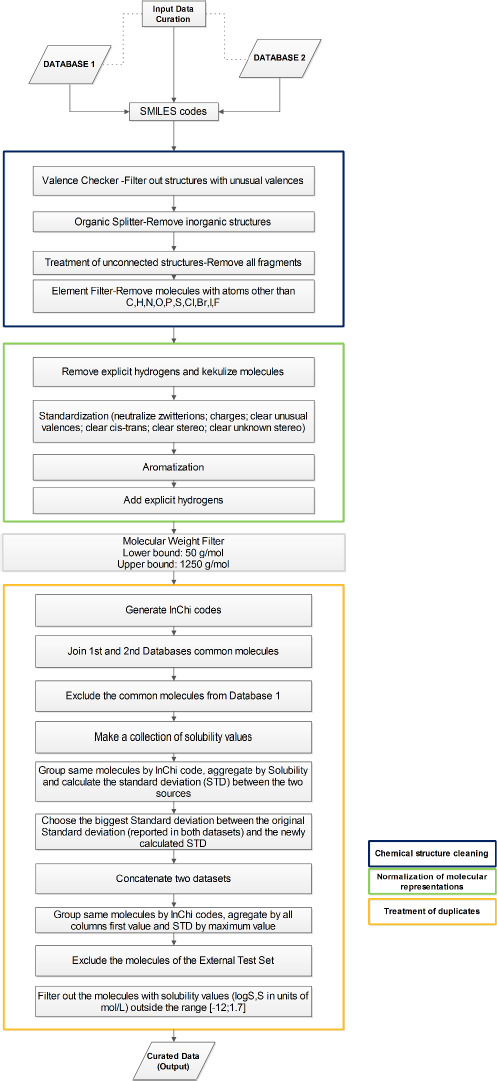
Protocol for data curation.

In order to create a single database containing only unique molecules matched to the most reliable aqueous solubility value, a general protocol for the curation of the database was developed. The curation protocol was divided into three parts: cleaning of chemical structures, standardization of the molecular representation and treatment of duplicates (see Figure 1).

The cleaning of the molecular structures followed the procedure described by Fourches *et al*. [25]. To apply this approach, a sequence of nodes was created in KNIME. First, the valence of the atoms was checked to filter out structures with unusual values. Subsequently, different filters were applied to maintain organic molecules, keep molecules containing only the following elements (C, N, O, H, F, Cl, Br, I, P and S), remove salts and unconnected molecules and filter out molecules out of weight bounds (between 50 and 1250 g/mol). Eventually the molecular representations were normalized and standardized.

For the treatment of duplicate structures, the first step was the generation of their *InChI* (International Chemical Identifier) code [26]. The duplicated molecules in both databases were identified and solubility values were collected. The *standard deviation values for the solubility measurements* (STD) were calculated and compared with the previous STD values recorded in both databases. To consider the variability in solubility measurement, the final reported value was the highest STD. Finally, these structures were concatenated with the unique molecules from both databases and care was taken to exclude from this set of molecules those belonging to the external test sets.

### Descriptor calculation

Molecular descriptors were calculated for all molecules using two KNIME extensions: the “*Descriptor”* node contained in the “*alvaDesc*” extension provided by *Alvascience Srl.* [23] and the *“RDKit Descriptor Calculation”* node contained in the RDKit extension [27]. In the first case, more than 1400 physicochemical descriptors (0D-2D) and molecular properties were calculated directly from molecular structure. The following families of descriptors were considered for modeling log *S*: constitutional indices, ring descriptors, topological indices, walk and path counts, connectivity indices, information indices, 2D matrix-based descriptors, 2D autocorrelations, Burden eigenvalues, P_VSA-like descriptors, ETA indices, functional group counts, charge descriptors and molecular properties. Another 45 molecular descriptors such as physicochemical properties, MOE-type and kappa descriptors, etc. were determined with *“RDKit Descriptor Calculation”.*

### Variable and data selection

#### Recursive variable selection

**Figure 2.**
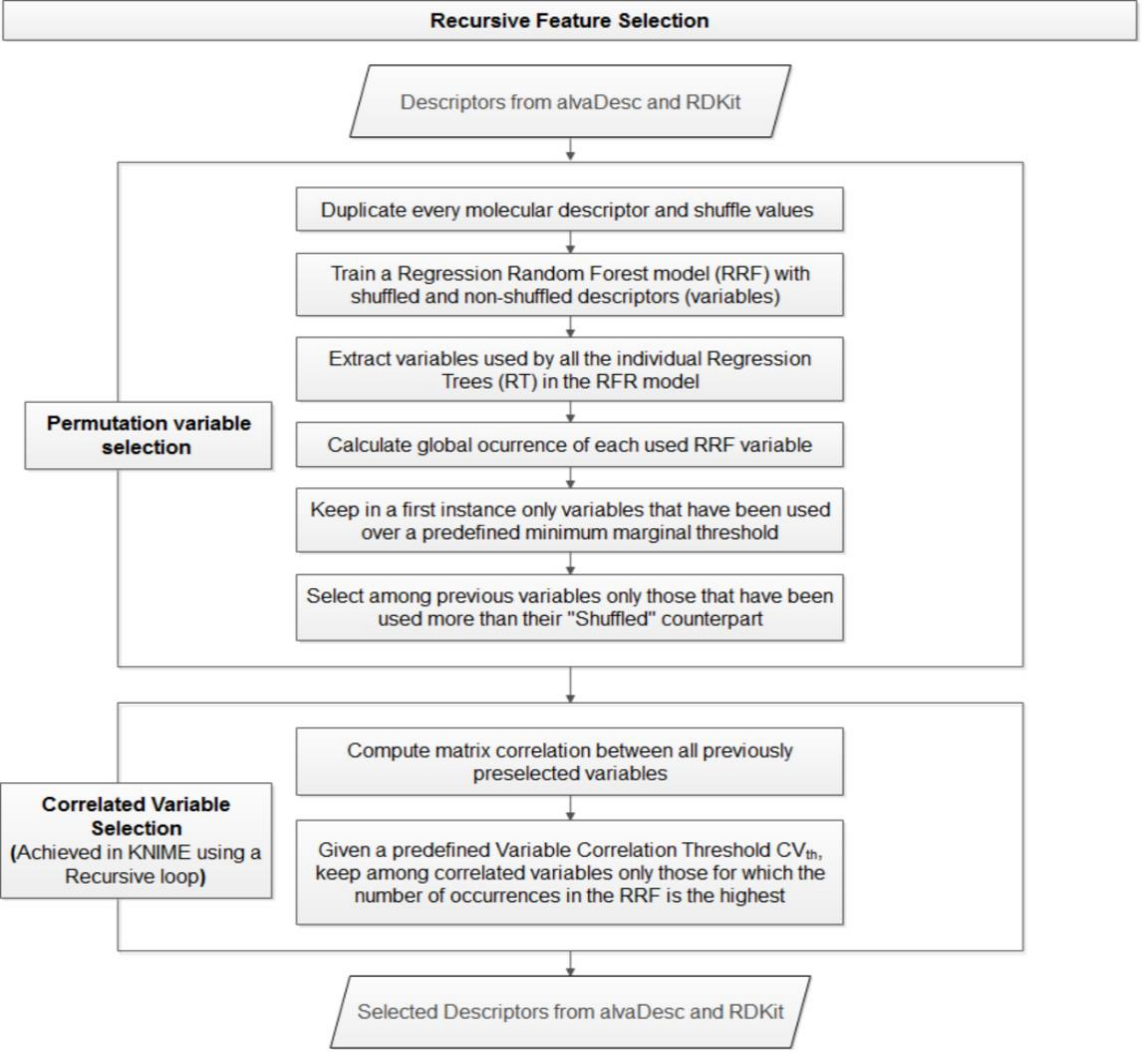
Schematic description of supervised recursive variable selection methodology

To determine the most relevant variables (descriptors) for the prediction of aqueous solubility, we developed a selection of variables by permutation using the Random Forest algorithm (RF), combined with a recursive selection of most correlated variables (see Figure 2). Numerical values of every molecular descriptor were shuffled and the RF model was trained with non-shuffled and shuffled counterpart variables according to the following parameters: the number of trees in the Forest (*ntree* = 11), the minimum branch node size (*nodesize* = 10) and bootstrap data sampling. Once the individual decision trees were extracted from the RF, the number of occurrences for each variable in the ensemble of trees was calculated, keeping only variables over a marginal threshold of occurrences (*threshold* = 110). Variables were retained at this step if the number of occurrences of the non-shuffled variable was greater than the number of occurrences of the counterpart shuffled variable. Finally, the number of variables was reduced in a recursive manner (recursive loop) by initially computing the linear correlation of the selected variables and then recursively selecting only those whose number of occurrences in the RF is greater than those of its correlated variables. In summary, only correlated variables with highest occurrence in the RF were eventually kept.

#### Recursive data cleaning

**Figure 3.**
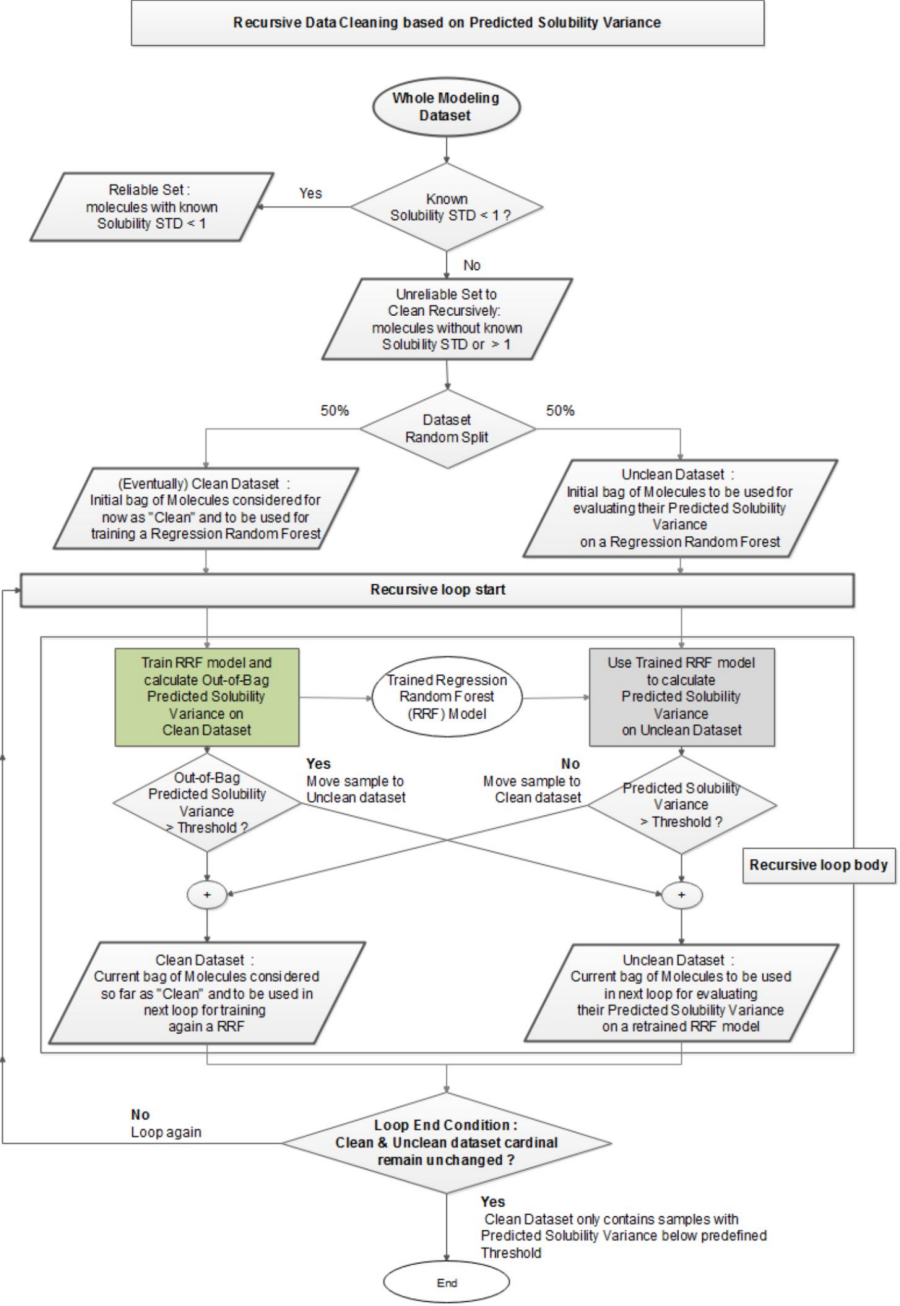
Schematic description of the supervised recursive data cleaning methodology

Considering the high variability of the experimental solubility measurements and the unreliable solubility data in several databases [28], a new recursive clean-up procedure was devised and carried out before the development of prediction models. In a first instance, all molecules in databases were organized as *RELIABLE*/*UNRELIABLE* according to their known *Solubility Standard Deviation* (STD) value. Molecules with known STD lower than 1 were considered to have good experimental solubility (*RELIABLE* set), while the remaining molecules with unknown STD (single measure) or STD ≥ 1 were considered to have high experimental variability (*UNRELIABLE* set). In the case of the *UNRELIABLE* data, a recursive procedure was carried out to clean this set. The dataset cleaning consisted of discriminating all the molecules that were systematically outside of an allowed threshold of *Predicted Solubility Variance* (PSV, *threshold* ≤ 1) during a training/classification-based process of selection. The PSV value is a measure of the variability of each individual prediction with respect to the average. Initially, this set of *UNRELIABLE* molecules was randomly partitioned into two 50 % and 50 % sets. Starting with one of the two partitions, a Regression Random Forest (RRF) was trained (*ntree* = 21, *tree depth* = 10 and *nodesize* = 10) and the RRF model was used to calculate the *Predicted Solubility Variance* of molecules in the other partition (out-of-bag estimator provided by KNIME RRF learner node). Through a recursive procedure, initially started from the first random partition, the molecules were either classified as within the PSV threshold (*CLEAN* data) or alternatively as beyond the PSV threshold (*UNCLEAN* data). This recursive process finished once the classification of molecules into the two sets stabilized after several iterations, which means that no molecules changed any more of categorized set, from *CLEAN* to *UNCLEAN or vice versa*. Eventually, a *CLEAN* set of low PSV is obtained, discarding all the molecules assigned to the *UNCLEAN* set. A detailed diagram of the procedure is shown in Figure 3.

### Selection of training and test sets

To evaluate the performance and stability of the *in-silico* solubility models, a split of the whole dataset was made based on the quality of the available solubility experimental data. From the global curated solubility dataset, initially partitioned and tagged as *RELIABLE* and *CLEANED* subsets (Figure 3), *CLEANED* data was used as the training set for the development of the model and *RELIABLE* data as a first test set. This sampling strategy ensures that the testing and evaluation of the model is based on the most reliable data without adding an optimistic bias on the training of the model when using for it the less reliable *CLEANED* data.

To validate the predictive ability of the models, an external set I of 181 molecules was used as an external validation. This external set was collected by Avdeef [29] and is made up of four publicly available sets with intrinsic solubility values reported. To further evaluate the workflow automatization, an external dataset II was also selected. To create this database, a search was carried out with the following keywords (*new molecules, experimental solubility, μM, and log S*). The final external set II eventually consisted of 30 new molecules reported in the last three years [30,31]. For these external evaluations and with the aim of increasing the methodology prediction performance on the external datasets, the *CLEAN* and *RELIABLE solubility subsets* were concatenated, and the model was retrained in this richer dataset to increase the structural diversity of the training set.

The supporting information includes a detailed list of the molecules belonging to each set as well as their solubility values (Tables S1, S2, S3 and S4, supplementary data).

### Model building and evaluation

**Figure 4.**
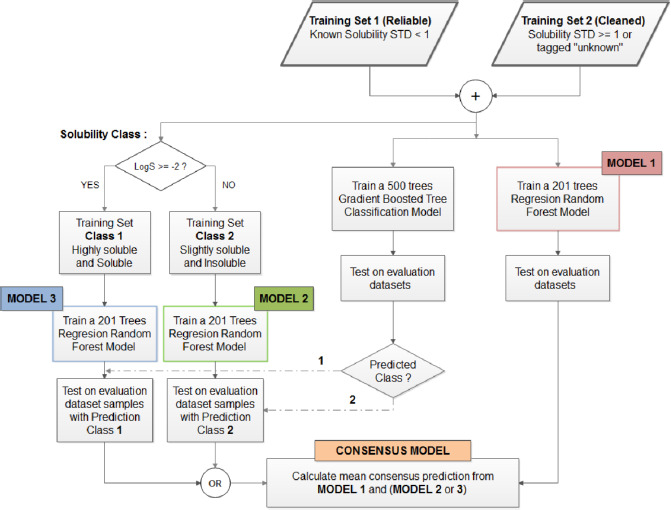
Schematic description of the modeling protocol

One of the main problems in estimating solubility is the fact that solubility values can vary over a wide range (several orders of magnitude). To develop local regression models based on two sections of the solubility range of our data set, we implemented a consensus model based on a combination of classification and regression techniques. The molecules belonging to *CLEANED* data were split into two distinct tagged classes according to log S values. Molecules with log *S* ≥ -2 (n = 4395) were tagged as soluble and highly soluble (Class 1), while molecules with log *S* < -2 (n = 7918) were tagged as slightly soluble and insoluble (Class 2). According to this rule, a Gradient Boosted Tree model was trained for classification. Two distinct regression models were developed to predict aqueous solubility for molecules belonging to separate Class 1 and Class 2 of the *CLEANED* data. This way, the range of solubility and structural diversity was restricted for each one of these sets. Both models were obtained using the *Tree Ensemble Learner (Regression)* node and their performance was evaluated using the molecules predicted as Class 1 and Class 2 by the Gradient Boosting method (see Figure 4 in the results section). It means that new test compounds are firstly classified in Class 1 or 2, by the Gradient Boosting method, and then according to this classification, they are predicted by one of the two regression models. In order to avoid the influence of a wrong classification due to border effects around the cut-off value, a third regression model was trained using all *CLEANED* data. Eventually, a consensus model was constructed, from the results of one of the local regression models and the regression model trained with all *CLEANED* data assuming the average log S estimated as the final value.

To demonstrate that the model can incorporate the information represented by the descriptors and relate it to solubility in the training set, the *RELIABLE* dataset was used as a test set. In order to increase the chemical diversity of the training set and obtain a better prediction of the external sets, the procedure described above was performed using the *CONCATENATED* data (*RELIABLE* + *CLEANED*) as the training set. Details on the consensus model are summarized in Figure 4.

Additionally, a classification model for aqueous solubility was carried out starting from the regression model by thresholding its output prediction (see Table 5). Considering that there isn’t a global consensus to select the cut-off value to be used in a binary classification of solubility, molecules with log *S* < -4 were regarded as insoluble in this study, while those with log *S* ≥ -4 were regarded as soluble. Similar cut-off values have been reported by other authors [12,32].

To assess the predictability of the regression and classification models in this study, a rigorous internal and external validation procedure was carried out following the guidelines of the Organization for Economic Co-operation and Development (OECD) [33]. The complete modeling and assessment process was included in a loop over five iterations to work out statistics with a view to demonstrate the consistency and robustness of the workflow.

To analyze the performance of the solubility regression models, some commonly used statistics were reported, including the regression coefficient of correlation squared (r^2^), the root mean squared error (RMSE) and the mean absolute error (MAE). The quality of the classification model was evaluated by analyzing the values of specificity (SP), sensitivity (SE), precision (PR), overall accuracy (OA), the Cohen's Kappa parameters and the area under the Receiver Operating Characteristic curve (AUC-ROC).

## Results and discussion

### Analysis of the new database of aqueous solubility

A new large and diverse database of aqueous solubility was generated. Once the database was carefully curated and tested, its solubility values ranged from -16 to + 2.1 logs units. However, the quality of this type of database with a wide range of solubility has been questioned [34]. According to Bergström and Larsson, the determination of concentrations of molecules with log *S* < -12 (picomolar) requires very sensitive analytical methods, while molecules with log *S* > + 1.7 (55, molar) means that these molecules are more soluble than the water in water, so the use of this type of data in QSPR models may affect their accuracy. In this sense, the final database of log S between -12 to +1.7 was composed of 12674 molecules. The statistical distribution on the log *S* scale is close to normal (skewness = -0.57 and excess kurtosis = 0.19) and contains a wide chemical diversity.

**Figure 5.**
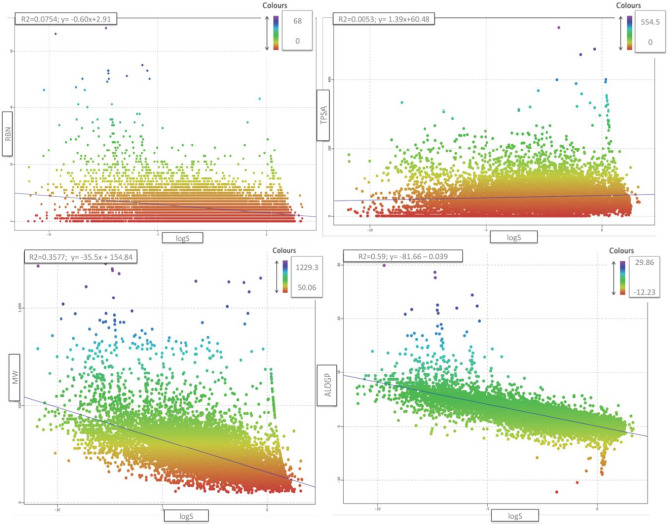
Correlation among RBN, TPSA, MW, and ALOGP properties and experimental aqueous solubility (log *S*). The colour scale refers to solubility ranges.

An analysis of the dataset on the distribution of properties in the chemical space defined by an *extended rule of five* (eRo5) shows its broad diversity and its potential use to develop models of solubility *in silico* with application during the processes of drug discovery and development. More than 99.5 % of all molecules are within the property range: molecular weight (*M*_W_ ≤ 1250 g/mol); topological polar surface area (TPSA ≤ 250); rotatable bonds (RBN ≤ 25); hydrogen bond donors (HBD ≤ 10) and hydrogen bond acceptors (HBA ≤ 15), and compounds such as antibiotics, antifungals, vitamins and cardiac glycosides that are initially outside the classical “*rule of five*” range are included in this dataset. The physicochemical properties of the database form a reasonable oral druggable space [35]. The distribution of the aqueous solubility data in the chemical space defined by some of these molecular properties is described in Table 1 and Figure 5.

As can be seen, properties such as *M*_W_, TPSA, RBN and ALOGP show clear trends with experimental solubility values. The best correlation was achieved with the ALOGP descriptor (r^2^=0.59), where a lower dispersion of this variable appears in the range of best solubility values (-5 < log *S* < 0). Outside this range there is an evident nonlinear correlation that appears for molecules with high solubility, very low ALOGP and high TPSA values (e.g. cyclodextrins, amikacins, sugars, etc.) and molecules with very poor solubility with extremely high ALOGP values, high number of RBN and large *M*_W_ (mainly molecules with large aliphatic chains). These results are justified because it is a composite variable related to steric and H-bonding interactions [36], which are quite important in the solubility of liquids and solid crystalline molecules.

The database was also analysed in terms of the quality of the solubility data. In this sense, 1839 molecules had reliable experimental data (Solubility STD ≤ 1) and it was called the *RELIABLE* dataset. Of the remaining molecules, with high variability data (Solubility STD > 1), a recursive cleaning procedure was able to identify 250 molecules with unreliable solubility data (in average after running statistics of five iterations on the whole process) and 10592 molecules formed the *CLEANED* dataset. Both datasets were *CONCATENATED* (12431 molecules) and were used to develop the regression and classification models of aqueous solubility for external datasets.

### Recursive variable and data selection

Variable selection by correlation is normally achieved as follows: For each candidate variable, the count of correlated variables is determined given a threshold value for the correlation coefficient. The variable with the highest number of correlated variables is kept and all its correlated variables are filtered out. This procedure is repeated until no more variables can be identified. This classical procedure of variable selection by correlation is made blindly without considering the relevance of every variable with respect to the variable being predicted, in this case solubility. This unsupervised strategy may lead to the elimination of variables which would have been selected as the most relevant during the construction of a decision tree model or more generally, of an ensemble of trees. In this work, we develop an alternative and new procedure for the supervised selection of variables using a recursive method based on a RF where we favor the variables which are the most explanatory to the variable being predicted, in this case solubility, as shown in Figure 2. Among more than 1400 molecular descriptors, the procedure identified in each of the five statistical iterations an average of 65 most significant variables and calculated the number of occurrences per variable, which allows categorizing their final relevance. After completing the statistics of five iterations, 138 most important descriptors were identified and the number of occurrences per variable was averaged. Table S5 lists all these variables as supporting information. This approach is quite relevant because it allows the most important variables to be categorized for final interpretation.

Similarly, a new recursive procedure was developed to clean the database of compounds with high and sometimes unreliable experimental aqueous solubility values [28]. The application of this recursive partitioning process resulted in a higher quality database of 10592 compounds, which was used to develop the solubility models and 243 molecules were identified as failed. After completing the statistics of five iterations, 615 molecules were highlighted as failed. Among these molecules, 57 were tagged as failed in all iterations. The results obtained with the use of this method, support its relevance, since we can rule out compounds potentially with extreme and variable solubility that affect the final prediction of the model.

### Predictive models for aqueous solubility

In order to find a way to improve the predictive accuracy of aqueous solubility models *in silico*, a new protocol was developed based on the combination of regression and classification models. To increase the performance of the individual models, a final consensus model was applied. A clear description of the modeling protocol is shown in Figure 4.

The predictive performance of the consensus model is summarized in Table 2. As can be seen in this table, the training set formed by the *CLEANED* dataset was able to adequately predict the solubility values of the test set (*RELIABLE* dataset). The r^2^= 0.87 in the test set is a good indicator of the model's ability to successfully predict new external compounds. For the four external sets (TS1-TS4), the predictions were quite different. The prediction of the external series with the model obtained with the *CLEANED* dataset was adequate for the TS1 and TS4 series (r^2^= 0.78-0.83, RMSE = 0.81-1.0) but not for TS2 and TS3 (r^2^= 0.40-0.47, RMSE = 0.98-1.02). However, once the model was trained with the *CONCATENATED* training set (*RELIABLE* + *CLEANED*), the statistics increased for all the series and especially for TS2 and TS3 (r^2^= 0.47-0.56, RMSE = 0.92-0.93).

**Figure 6.**
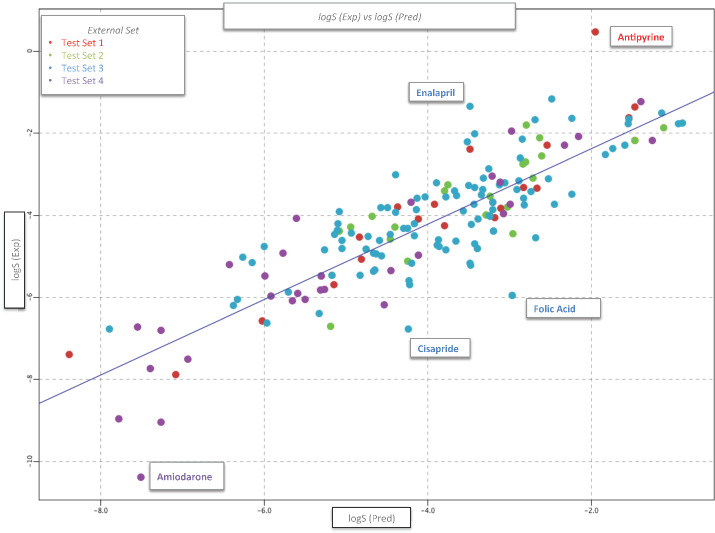
Prediction of aqueous solubility (log *S*) for the external set I, using the final consensus model

Eight drugs (4.4 % of the external set I) with standardized residues greater than 2 or less than -2 were identified as response outliers. They were folic acid (TS2 and TS3), antipyrine (TS1), amiodarone (TS4), cisapride (TS3), enalapril (TS3), rifabutin (TS4), ritonavir (TS3) and mifepristone (TS4). From these compounds, only five were identified as outliers in all iterations (see Figure 6).

Compounds such as antipyrine and mifepristone have been also erroneously predicted by other authors [15,29]. It is described that these drugs have conflicting solubility data reported in the literature, and in the case of antipyrine, multiple and different experimental solubility values (-0.66 to 0.55) have been reported [29,37,38]. The wrong prediction of compounds such as amiodarone and mifepristone has been attributed to extreme solubility values which made their detection more difficult with sensible analytical methods and sometimes, to obtain the complete solubilization, requires the use of co-solvents [38]. In the case of compounds with very high solubility (antipyrine) and very low solubility (amiodarone and mifepristone), their solubility prediction could be influenced by a poor representation of similar compounds in the chemical space of the database [39,40]. Folic acid has been poorly predicted by other authors [29,40], which has conflicting solubility data reported in the literature attributed to drug degradation. The low representation of high solubility compounds within the database (7 %) could influence the large prediction error for the Enalapril.

However, the solubility values reported for external set I were intrinsic solubility values, while the consensus model was developed with molecules with aqueous solubility. This suggests, that in the first case the value corresponds to the solubility of a compound in its free acid or base form at a pH where it is completely non-ionized, and in the second case the solubility depends on the pH used to perform the measurements and therefore can be diﬀerent for ionizable compounds. In general, the aqueous solubility is greater than or equal to the intrinsic solubility [2]. Considering the above, the intrinsic solubility values for folic acid, amiodarone, enalapril, antipyrine and cisapride were replaced by aqueous solubility data collected in different publications (see Table 3). For folic acid, amiodarone and cisapride our model predicts values higher than the reported intrinsic solubility value, and closer to the experimental aqueous solubility (pH around 6.8) reported in the literature. In the case of enalapril, the reported intrinsic solubility value is for enalapril maleate and not for the free base (enalapril), in this sense our prediction is closer to the free base (input structure). Considering the variability described for antipyrine, we found that the value predicted by our model was close to other reported experimental values. Finally, the prediction of the external set I improved significantly, considering the new aqueous solubility values for these five compounds, with better results for series TS1, TS2 and TS4 (r^2^= 0.72-0.87, RMSE = 0.65-0.79), and the lowest prediction was for set TS3 (r^2^= 0.51, RMSE = 0.85).

The prediction of aqueous solubility for all datasets of the external set I was also evaluated using Pipeline Pilot software [43] (see Table 4). The aqueous solubility Pipeline Pilot algorithm is built-in and provided as a “black box” node for prediction only, with no possibility of training or parameterization. The algorithm is based on Tetko *et al*. method [44]. The results obtained demonstrate the difficulty of predicting these data sets. The predictions of our consensus model (see Table 2) are satisfactory in comparison with these results, which reinforces the potential of the methodology developed and the predictive capacity of the final model obtained.

Chevillad *et al*. [8] showed the performance of solubility predictions for different commercial software on five data sets. Table 5 shows the comparison between the results described in this study and our results for the Test Set 2 (in this paper), originally used in the first Solubility Challenge. The results obtained in our proposal (r^2^= 0.56, RMSE = 0.93) suggest a good performance compared to the models with the best individual predictions (Avdeef and QikProp-CI). However, when the intrinsic solubility value of the folic acid (one of the five outliers detected for all external set I) was replaced by the aqueous solubility value, the results significantly improved (r^2^= 0.72, RMSE = 0.73).

**Figure 7.**
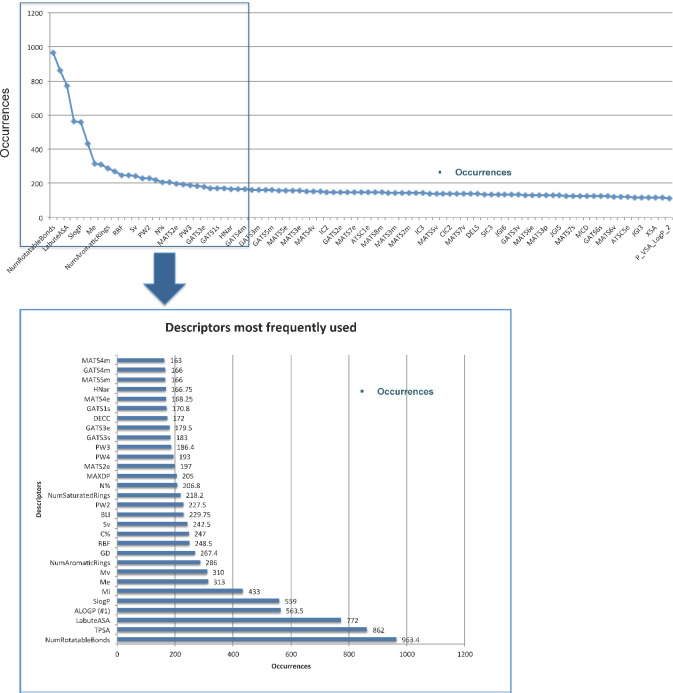
Descriptors most frequently used in the prediction of aqueous solubility (log S).

Figure 7 shows an analysis of the occurrences of 138 variables selected by *Supervised Recursive Variable Selection* after completing the five iterations of our modeling workflow. The most frequent descriptors were ranked by their importance (only the first 30 descriptors are shown), which means that only a few of the descriptors contribute significantly to the model. Among the most frequent descriptors are variables related to hydrophobicity (partition coefficients such as ALOGP and SlogP), steric (number of aromatic rings, number of saturated rings, etc.), hydrogen bonding (Labute´s Approximate Surface Area, topological polar surface area, etc.), molecular flexibility (number of rotatable bonds, rotatable bond fraction, etc.), electrostatic (mean atomic Sanderson electronegativity, mean first ionization potentials) and topological interactions [45]. Most of these descriptors have been highlighted by other authors for their correlation with solubility [29] [46]. In other cases, they show a high correlation.

Table 6 summarizes the statistical results of the classification model that was carried out starting from the regression model by thresholding its output prediction. As can be seen from this table, the model had excellent balanced accuracy and Cohen’s Kappa values for training and internal test sets. Moreover, the sensitivity values or percentage of high solubility molecules correctly predicted, for training and internal test sets, were quite similar to the specificity values or percentage of low solubility molecules.

The model had very good performance in the prediction of the external set I, with accuracy values between 74-96 % and Cohen’s Kappa between 0.46-0.86. The ROC curve analysis corroborated the good performance of the model with areas under curve of 99 %, 96 % and 91 % for training, internal test and external test sets, respectively. All the ROC curves are depicted in Figure S1 of supporting information.

### Automatic workflow for the prediction of aqueous solubility

**Figure 8.**
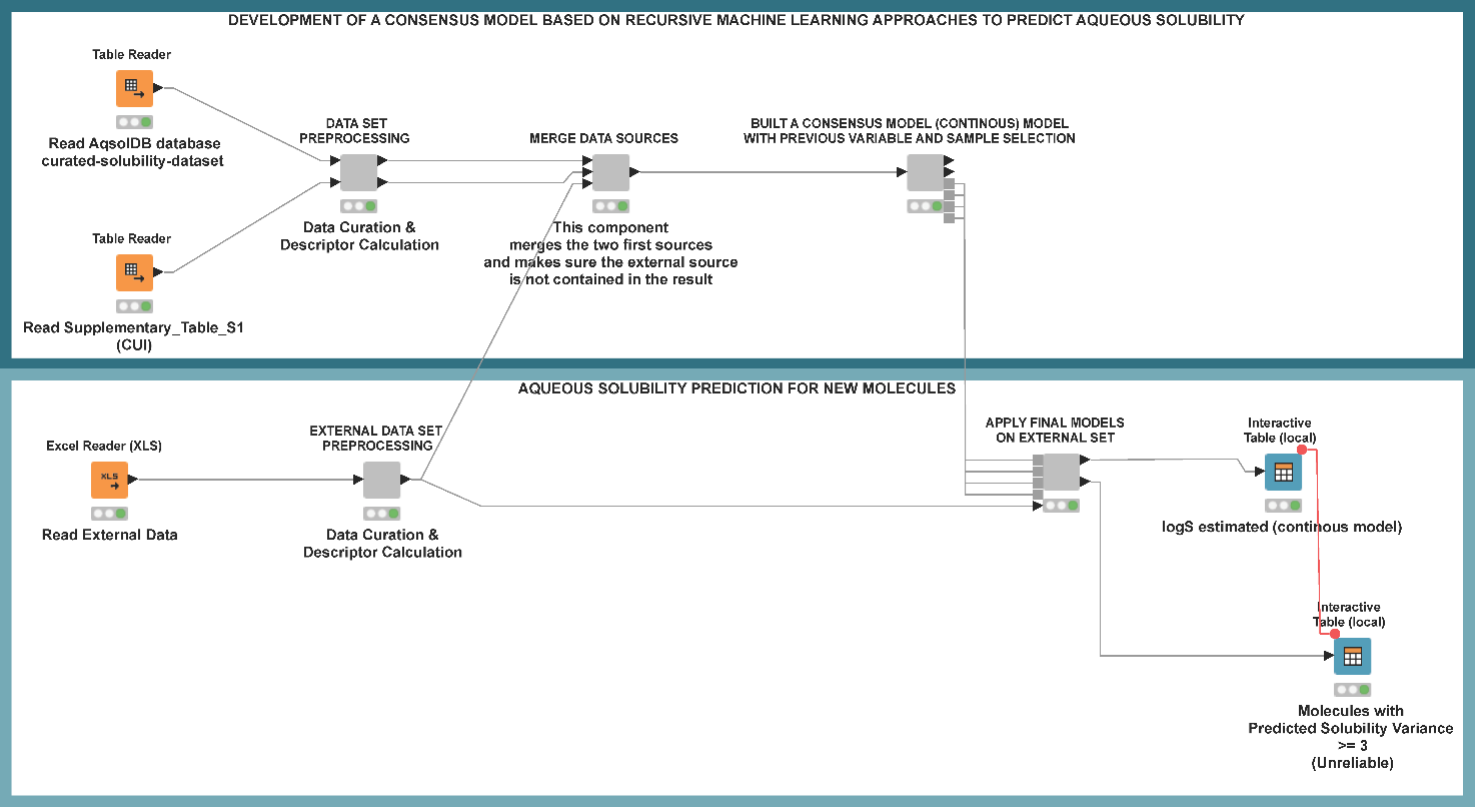
Workflow for modeling aqueous solubility. From top to bottom. First rectangle: Development and validation of QSPR protocol; second rectangle: the same sequence of steps to automate the prediction of a new external dataset based on the consensus model obtained in the development section.

The final workflow integrated two main sections (see Figure 8). The first one was related to the development of the QSPR protocol and included the preprocessing of the data (data curation, parametrization of the molecular structure, etc.), the merging of the data sources (combination of different datasets) and the development of regression and consensus models (recursive selection of variables and data cleaning, data partition, building, training and validation of the consensus model). The second section was designed to automate the prediction of a new external set based on the consensus model obtained in the preliminary development section. In order to validate the functionality of this workflow, a new dataset made of 30 new molecules from the last three years was assembled. As a method to identify unreliable predictions and in accordance with the recursive data cleaning developed algorithm, we proposed to take *Solubility Prediction Variance* ≥ 3 as a criterion to highlight the molecules with suspect predictions. The results reached for this External set II are shown in Table 7.

### Comparison with previous in silico solubility models

Several QSPR models have been previously published to predict aqueous solubility. All models published in the last ten years and their performances are clearly described in Table 9.

Among regression models, only one was derived using large datasets (over 8000 compounds). Cui *et al*. [12] developed a deep neural network (DNN). However, their prediction of the external set was low in terms of r squared but good for RMSE (r^2^= 0.39, RMSE= 0.68). As far as we know, this study generated one of the largest aqueous solubility data with more than 12000 compounds. The overall prediction for all compounds belonging to the external set I (ALL) is good (r^2^= 0.69, RMSE= 0.92), but once the intrinsic solubility values of the five outliers were replaced by the aqueous solubility values, the results improved (r^2^= 0.73, RMSE = 0.80).

For our classification model, the overall results showed a better performance in predicting the soluble and insoluble classes compared to other published models. One of the most widely used external test sets was 32 molecules of the first solubility challenge. In this dataset our model had a balanced accuracy of 86 %, with a specificity value of 71 % and sensitivity value of 100 %, highlighting a better performance of our proposal.

## Conclusions

This study reported the first automatic workflow, developed on the KNIME Analytics Platform, to predict aqueous solubility of compounds. All steps of the QSPR were automated, focusing on data integration and curation procedures to obtain an extensive and structurally diverse database of aqueous solubility. Based on the large set of solubility data, the relationships between four simple molecular properties and solubility have been studied. By combining two recursive machine learning approaches for data cleaning and variable selection, and two regression and classification algorithms in a consensus model, good regression and classification statistics were obtained to predict the aqueous solubility of molecules. However, these results could be improved by increasing the chemical space of our dataset with more drugs and more reliable samples. The workflow developed was designed to automate the prediction of aqueous solubility of new databases, starting from chemical structure. These results highlight the relevance of this model, during the early stage of drug discovery and development.

## Figures and Tables

**Table 1.** Distribution of aqueous solubility data in the chemical space defined by six molecular properties

Solubility Class				
Physicochemical descriptor	log *S* ≥ 0 (Highly soluble)	log *S* < 0 and log *S* ≥ -2 (Soluble)	log *S* < -2 and log *S* ≥ -4 (Slightly soluble)	log *S* < -4 (insoluble)
*M*_W_ *Mean (Std)*	159.0 (95.3)	191.2 (83.5)	249.5 (89.00)	348.7 (134.4)
TPSA *Mean (Std)*	62.4 (51.5)	57.7 (40.1)	58.1 (38.1)	51.1 (43.9)
ALOGP *Mean (Std)*	-0.3 (1.5)	1.0 (1.2)	2.2 (1.2)	4.7 (2.5)
RBN *Mean (Std)*	2.8 (3.1)	2.9 (2.7)	4.0 (3.3)	6.0 (6.6)
HBD *Mean (Std)*	1.8 (2.1)	1.3 (1.4)	1.1 (1.2)	0.8 (1.2)
HBA *Mean (Std)*	3.5 (2.8)	3.3 (2.3)	3.5 (2.2)	3.5 (2.7)
**N. compounds**	924	3519	4710	3521
**% of data**	7	28	37	28

**Table 2.** Performance of the final consensus model for the training, internal test and external test sets

**Training (*CLEANED*)**						
**Dataset**	**r^2^**		**RMSE**		**MAE**	
	**Mean**	**Std**	**Mean**	**Std**	**Mean**	**Std**
**Training Set**	0.97	0.00	0.39	0.00	0.25	0.00
**Internal Test Set (*RELIABLE*)**	0.87	0.00	0.75	0.01	0.55	0.01
**External Set I (All)**	0.64	0.02	0.97	0.03	0.77	0.01
**TS1**	0.83	0.02	0.81	0.06	0.62	0.05
**TS2**	0.47	0.03	1.02	0.03	0.78	0.02
**TS3**	0.40	0.03	0.98	0.02	0.78	0.02
**TS4**	0.78	0.04	1.00	0.08	0.81	0.05
***CONCATENATED* training (*CLEANED* + *RELIABLE*)**						
	**r^2^**		**RMSE**		**MAE**	
	**Mean**	**Std**	**Mean**	**Std**	**Mean**	**Std**
**External Set I (All)**	0.69	0.02	0.92	0.03	0.71	0.02
**TS1**	0.84	0.02	0.79	0.04	0.60	0.01
**TS2**	0.56	0.04	0.93	0.04	0.70	0.03
**TS3**	0.47	0.03	0.92	0.03	0.73	0.02
**TS4**	0.80	0.02	0.96	0.04	0.74	0.03

r^2^: the squared of correlation coefficient of regression, Std: standard deviation obtained from a five iterations loop, RMSE: root mean squared error, MAE: mean absolute error. TS1-TS4: test set 1 to 4.

**Table 3.** Aqueous solubility values for the five outlier compounds detected with the consensus model

Compound	Log S^a^_exp_	Log S^b^_pred_	Log S^c^_exp_	Reference
Folic acid	-5.96	-2.96	> -2.87	[41]
Antipyrine	0.45	-1.95	-0.58	[42]
Amiodarone	-10.4	-7.51	< -7.17	[42]
Cisapride	-6.78	-4.23	-4.7	[42]
Enalapril	-1.36	-3.48	-3.33	[4]

^a^experimental intrinsic solubility values [29];

^b^aqueous solubility predicted with the consensus model;

^c^experimental aqueous solubility values collected from literature.

**Table 4.** Performance of Pipeline Pilot (PP) in the prediction of aqueous solubility for the External Set I (N=181)

External Set I	r^2^	RMSE	MAE
**TS1**	0.73	1.01	0.79
**TS2**	0.08	1.34	1.03
**TS3**	-0.71	1.65	1.12
**TS4**	0.45	1.59	1.12
**All data**	0.12	1.53	1.07

r^2^: the squared of correlation coefficient of regression, RMSE: root mean squared error, MAE: mean absolute error. TS1-TS4: test set 1 to 4

**Table 5.** Performance of different models published in the prediction of our Test Set 2 (belonging to Solubility Challenge)

	Solubility Challenge (N = 26)	
Models	r^2^	RMSE
PP-solubility	0.29	1.66
PP-ADMET solubility	0.28	1.49
ACD	0.53	1.14
MOE	0.44	1.30
QikProp	0.34	1.24
QikProp-CI	0.57	1.12
ADMET PREDICTOR	0.32	1.25
Volsurf +	0.44	1.09
FAF	0.18	1.19
VCC lab	0.39	1.18
ISIDA	0.35	1.09
RFR [29]^a^	0.57^b^	0.92^b^
This study^a^	0.56	0.93
	0.72^c^	0.73^c^

^a^ In these studies, 28 compounds were included in the second dataset.

^b^ Values calculated from the original data reported in the article (Table A2) [29].

^c^ Results obtained when the intrinsic solubility value of folic acid (outlier) was replaced by the aqueous solubility value. PP: Pipeline Pilot

**Table 6.** Performance of the classification model for the training, internal test and external test sets

**Training (*CLEANED*)**								
	**Balanced Accuracy**		**Cohen´s Kappa**		**Sensitivity**		**Specificity**	
	**Mean**	**Std**	**Mean**	**Std**	**Mean**	**Std**	**Mean**	**Std**
**Training Set**	0.94	0.00	0.90	0.01	0.98	0.01	0.92	0.01
**Internal Test Set (*RELIABLE*)**	0.88	0.01	0.78	0.01	0.96	0.01	0.80	0.01
**External Set I (All)**	0.80	0.01	0.60	0.04	0.89	0.01	0.71	0.02
**TS1**	0.85	0.01	0.61	0.01	0.83	0.01	0.78	0.01
**TS2**	0.79	0.01	0.71	0.01	1.00	0.01	0.71	0.01
**TS3**	0.74	0.01	0.46	0.05	0.85	0.01	0.61	0.01
**TS4**	0.96	0.01	0.86	0.01	0.91	0.01	0.91	0.01
***CONCATENATED* training (*CLEANED* + *RELIABLE*)**								
	**Balanced Accuracy**		**Cohen´s Kappa**		**Sensitivity**		**Specificity**	
	**Mean**	**Std**	**Mean**	**Std**	**Mean**	**Std**	**Std**	**Std**
**External Set I (All)**	0.81	0.01	0.61	0.04	0.92	0.01	0.70	0.01
**TS1**	0.85	0.01	0.70	0.01	0.92	0.01	0.78	0.01
**TS2**	0.86	0.01	0.71	0.01	1.0	0.01	0.71	0.01
**TS3**	0.76	0.01	0.50	0.05	0.88	0.02	0.64	0.01
**TS4**	0.91	0.01	0.74	0.01	1.00	0.01	0.82	0.01

Std: standard deviation obtained from a five-iteration loop, RMSE: root mean squared error. TS1-TS4: test set 1 to 4.

**Table 7.** Performance of the final consensus model for the external set II (N=30)

*CONCATENATED* training (*RELIABLE+CLEANED*)						
	r^2^		RMSE		MAE	
External Set II	Mean	Std	Mean	Std	Mean	Std
**All data**	0.43	0.01	0.73	0.02	0.56	0.01
**Data without outliers (n=27)^a^**	0.66	0.01	0.59	0.01	0.46	0.01

r^2^: the squared of correlation coefficient of regression, Std: standard deviation obtained from a five iteration loop, RMSE: root mean squared error, MAE: mean absolute error.

^a^ Molecules with Solubility Prediction Variance < 3.

**Table 8.** Performance of the classification model for the external set II (N=30)

**Training (*CLEANED*)**								
	**Balanced Accuracy**		**Cohen´s Kappa**		**Sensitivity**		**Specificity**	
	**Mean**	**Std**	**Mean**	**Std**	**Mean**	**Std**	**Mean**	**Std**
**External Set II**	0.83	0.01	0.67	0.01	0.73	0.01	0.93	0.01
***CONCATENATED* training (*CLEANED* + *RELIABLE*)**								
	**Balanced Accuracy**		**Cohen´s Kappa**		**Sensitivity**		**Specificity**	
	**Mean**	**Std**	**Mean**	**Std**	**Mean**	**Std**	**Mean**	**Std**
**External Set II**	0.80	0.01	0.60	0.02	0.73	0.01	0.87	0.01

Std: standard deviation obtained from a five iteration loop, RMSE: root mean squared error.

**Table 9.** Summary of the QSPR models (Regression and Classification) for aqueous solubility prediction published in the last ten years

Ref.	Model type	Method	N_train_	N_test_	N_ext_	Descriptors	Better Model performance for external sets
[47]	Regression	MLR, ANN and SVM	60	14		Topological and structural descriptors	ANN: *R*_test_ = 0.77, RMSE_test_= 0.74 SVM: R_test_ = 0.731, RMSEtest= 0.83
[48]	Classification	DT, NB, NN, SVM and DF	762	412	102	1D-3D descriptors and fingerprints	T_set_: LS (86.7 %), MS (83.9 %), HS (88.7 %) V_set_: LS (71.9 %), MS (60.6 %), HS (71.6 %) E_set_: LS (43.3 %), MS (54.2 %), HS (60 %)
[49]	Classification	RF, SVM, BRANNs	711	131	T1:747 T2: 32	pharmacophore fingerprint, MOE, Volsurf and ParaSurf08 descriptors	External test set 1(accuracy): 64.7 % (three classes) External test set 2 (accuracy): 77.8 %
[32]	Classification	SVM	41501	4510	32	Fingerprints and physicochemical properties	Test set (accuracy): 84 % External test set (accuracy): 78.1 % Sensitivity: 88 %; Specificity: 43 %
[8]	Regression	RF	3970		D1: 26 D2:150	Fingerprints	D2: *R*_test_ = 0.84, RMSE_test_= 0.71
[50]	Regression	PLS	1004	252		Log P, TPSA and melting point	*R^2^*_test_ = 0.87, RMSE_test_= 0.61
[11]	Regression	RNN	D1: 1029 D2: 923 D3: 60 D4: 97	D1: 115 D2: 103 D3: 14 D4: 28		Feature vectors	R^2^ = 0.92; RMSE = 0.58 R^2^ = 0.91; RMSE = 0.60 R^2^ = 0.81; RMSE = 0.58 R^2^ = 0.67*; RMSE = 0.90*
[41]	Regression	MLR, MLREM, BRANNLP	3567	911	32	86 Volsurf descriptors	MLR: *R^2^*_test_ = 0.89, SEP = 0.75 MLREM: R^2^_test_ = 0.88, SEP = 0.76; R^2^_ext_ = 0.81, SEP = 0.75 BRANNLP: R^2^_test_ = 0.90, SEP = 0.66; R^2^_ext_ = 0.86, SEP = 0.65
[36]	Regression	AMP, LoRep	2093	522	43	32 HYBOT and DRAGON descriptors (2 descriptors)	*R^2^*_test_ = 0.86-0.94, SD= 0.59-0.85 R^2^_ext_ = 0.62-0.70, SD= 0.58-0.89
[51]	Classification/ Regression	Global: MLR, RF, SVM Local: RCNN, AMP, LoReP	D1: 818 D2: 2093 D3: D1+D2	D1: 204 D2: 522 D3: D1+D2		12 2D-physicochemical descriptors	D1: Consensus: R^2^_test_ = 0.93; RMSE = 0.58 D2: Consensus: R^2^_test_ = 0.88; RMSE = 0.80 D3: Consensus**:** R^2^_test_ = 0.88; RMSE = 0.81
[46]	Regression	Global: MLR, RF, SVM Local: RCNN, AMP, LoReP	349	38		15 physicochemical descriptors (HYBOT, DRAGON, SYBYL and VolSurf+)	RF: R^2^_test_ = 0.74; RMSE = 0.72 SVM: R^2^_test_ = 0.71; RMSE = 0.76 AMP: R^2^_test_ = 0.75; RMSE = 0.70 LoReP: R^2^_test_ = 0.73; RMSE = 0.73 Consensus: R^2^_test_ = 0.79; RMSE = 0.64
[52]	Regression	CNN	1116			Fingerprints	R2 = 0.93; RMSE = 0.56
[14]	Regression	RF, GBDT, MT-DNN	1708		D1:1207 D2:21 D3:120	Topological and 2D descriptors	MT-ESTD R^2^_test-1_ = 0.94; RMSE = 0.69 MT-ESTD R^2^_test-2_ = 0.97; RMSE = 0.65
[12]	Regression	DNN	8949	994	62	RDKit descriptors	R^2^_ext_ = 0.39; RMSE = 0.68
[29]	Regression	MLR, RF	4449	196	D1: 21 D2: 28 D3: 100 D4: 32	RDKit and ABSOLV descriptors	*R^2^_ext-1_ = 0.83; RMSE = 0.84* *R^2^_ext-2_ = 0.57; RMSE = 0.92* *R^2^_ext-3_ = 0.66; RMSE = 0.75* *R^2^_ext-4_ = 0.77; RMSE = 1.05*
This study	Regression/ Classification	RF, GBT	T2: 12431	1834		65 2D-descriptors (alvaDesc, RDKit)	Consensus model: R^2^_IT_ = 0.87; RMSE = 0.74
			T1: 10592		Ext I T1: 21 T2: 28 T3: 100 T4: 32 Ext II: 30	65 2D-descriptors (alvaDesc, RDKit)	Consensus model: r^2^Ext I = 0.69; RMSE = 0.92 (r^2^Ext I = 0.73; RMSE = 0.80)^a^ r^2^_t1_ = 0.84; RMSE = 0.79 (r^2^_t1_ = 0.88; RMSE = 0.65)^a^ r^2^_t2_ = 0.56; RMSE = 0.93 (r^2^_t2_ = 0.72; RMSE = 0.73)^a^ r^2^_t3_ = 0.47; RMSE = 0.92 (r^2^_t3_ = 0.51; RMSE = 0.85)^a^ r^2^_t4_ = 0.80; RMSE = 0.96 (r^2^_t4_ = 0.83; RMSE = 0.79)^a^ r^2^Ext II= 0.66; RMSE: 0.55 Classification model (accuracy): Ext I = 0.83 % Sensitivity: 89 %; Specificity: 71 %

ANN: Artificial neural network; SVM: Support vector machines; MLR: Multilinear regression; RF: Random Forest; PLS: Partial Least-Squares; RNN: Recursive Neural Networks; MLREM: multiple linear regression employing an expectation maximization algorithm and a sparse prior method; BRANNLP: Bayesian regularized artificial neural network with a Laplacian prior; AMP: arithmetic mean property; LoRep: local one-parameter regression. RCNN: regression corrected by nearest neighbors; AMP: arithmetic mean property and LoReP: local regression property. GBDT: Gradient boosting decision tree. MT-DNN: Multitask deep neural network; DNN: Deep neural network; GBT: Gradient Boosting Tree; DT: Decision Tree; NB: Naïve Bayes; NN: Neural Network; DF: Decision Forest;

^a^Results obtained when the intrinsic solubility values of the five outliers were replaced by the aqueous solubility values.

## Abbreviations

RMSE: the root mean squared error. RMSE = [ 1/n Σ_i_ (*y*_i_^obs^ – *y*_i_^calc^)^2^ ]^1/2^, where *y*^obs^/ *y*^calc^ = observed/calculated value of log *S* according to model, n = number of measurements of log *S*.
r^2^: the squared of correlation coefficient of regression. r^2^ = 1 - Σ_i_ (*y*_i_^obs^ - *y*_i_^calc^)^2^ / Σi (*y*_i_^obs^ - <*y*>)^2^, where *y*=log *S*, and <*y*> is the mean value of log *S*.
MAE: mean absolute error. MAE = 1/n Σ_i_ | *y*_i_^cal^ – *y*_i_^obs^|
SE: sensitivity. SE = TP / (TP+FN)
SP: specificity. SP = TN / (TN+FP)
OA: overall accuracy. OA = (TP+TN) / (TP+FP+TN+FN)
PR: precision. PR = TP / (TP+FP)
Cohen's Kappa: (OA-EA) / (1-EA), where EA = ((TP+FP) (TP+FN)+(TN+FP)(TN+FN)) / (TP+FN+FP+TN)^2^
TP: true positives
TN: true negatives
FP: false positives
FN: false negatives
EA: the expected accuracy based on the marginal totals of the confusion matrix
ntree: number of trees in the random forest approach
nodesize: the minimum branch node size in the random forest approach
